# Smoking-attributable burden of lung cancer in Mongolia a data synthesis study on differences between men and women

**DOI:** 10.1371/journal.pone.0229090

**Published:** 2020-02-14

**Authors:** Ariuntuya Tuvdendorj, Talitha Feenstra, Badamsuren Tseveen, Erik Buskens

**Affiliations:** 1 Department of Health Policy, School of Public Health, Mongolian National University of Medical Sciences, Ulaanbaatar, Mongolia; 2 Department of Epidemiology, University Medical Center Groningen, University of Groningen, Groningen, the Netherlands; 3 Center for Nutrition, Prevention and Health Services, National Institute for Public Health and the Environment (RIVM), Bilthoven, the Netherlands; 4 National Cancer Center, Research Training and Information Department, Ulaanbaatar, Mongolia; National Health Research Institutes, TAIWAN

## Abstract

**Background:**

Smoking is widely recognized as one of the most prevalent and preventable causes of many cancer types. This study aimed to quantify the population attributable fraction (PAF) of the lung cancer burden for smoking in Mongolia.

**Methods:**

Lung cancer incidence and lung cancer-related death data came from the population-based national registry covering the period 2007–2016. Smoking prevalence data came from the STEPwise approach (STEP) national survey. The lung cancer-related disease burden was calculated and expressed in Disability Adjusted Life Years (DALYs) lost by gender and by year. This was combined with current smoking and former smoking prevalence data, and relative risks (RR) of lung cancer-related deaths for current smokers and former smokers versus never smokers from region-specific cohort studies to estimate the PAF of lung cancer attributable to “ever-smoking” in Mongolia.

**Results:**

Between 2007 and 2016, lung cancer accounted for the loss of over 63,000 DALYs in Mongolia. The PAF of lung cancer-related deaths attributable to current and former smoking combined was 58.1% (95% IR = 43.1%-72.2%) for men and 8.9% (95% IR = 4.1% -13.5%) for women. Smoking-attributable DALYs loss amounted to 2589 years (95% IR = 1907–3226) in 2016.

**Conclusions:**

A considerable health loss may be prevented with an effective anti-smoking policy. In Mongolia, more than one third of lung cancer-related DALY loss is attributable to active smoking, and thus is potentially preventable. Furthermore, a gender-specific tobacco control policy may be worthwhile because of the large gender difference in smoking exposure in Mongolia. Next to this, age specific policy, including a smoke–free generation policy for adolescents, with targeted education, and mass media campaigns is needed.

## Introduction

Smoking is a major risk factor for many cancer types [1]. It is the second largest contributor to the global disease burden, accounting for 148.6 million disability-adjusted life years (DALYs) lost in 2015 [2]. Globally, almost 70% of all lung cancer deaths among adults are attributable to smoking [3].

Although tobacco smoking is the most established risk factor for lung cancer, the effects of smoking used to be larger in Western countries than Asian countries [4,5]. A comprehensive review found high relative risks in North America and low relative risks in Asia [6]. In the US the population attributable fraction (PAF) of lung cancer deaths caused by smoking was 90% for men and 87% for women [7]. In contrast, in Asia this was 60% for men and 17% for women [8]. The lower risk of smoking-related lung cancer death in Asian countries may be explained by the different socioeconomic conditions, genetic susceptibility and different smoking behaviours in these countries [9]. Many Asian countries are in earlier phases of the smoking epidemic than most Western high income countries [10, 11].

In Mongolia, smoking prevalence has remained unchanged in the past ten years [12][13][14]. Almost 50% of men and 5% of women aged 15 years and over were smokers. Epidemiological evidence shows that smoking-related diseases have increased over time [15]. Lung cancer mortality has been one of the five most common cancers in recent years [16]. In particularly, the age-standardized rate of lung cancer death (per 100,000 persons-years) was substantially higher in men (36.9) than in women (7.0) [17].

While landmark international studies have revealed the effect of smoking for every country and region, these studies applied the same relative risks (RR) of smoking to all countries [2][3][18]. In other words, they did not distinguish between countries or cultures. The estimate for relative risk was taken from the American Cancer Society Cancer Prevention Study-II (CPS-II), where most current smokers were life-long Caucasian cigarette smokers with a mean consumption of about twenty cigarettes per day [19]. In contrast, in Mongolia, the mean number of cigarette smoked per day was reported to be 10,3 for men and 6.4 for women with a mean duration of smoking of 21 years for men and 19 years for women [14]. Given the low smoking intensity and relatively short smoking duration, it seems more appropriate to use a region-specific RR.

This is the first attempt to estimate the magnitude of the burden caused by tobacco smoking on population health in Mongolia, in particular for lung cancer. Mongolian results were part of global comparison studies [2][3][18]. In addition to using less suitable RRs, based on Western studies, most of these studies used the indirect method developed by Peto and Lopez [2][3][20]. This indirect method uses the lung cancer mortality as a proxy for smoking prevalence. Moreover, they applied the method based on the estimated lung cancer death rates among smokers and never-smokers in American populations. Given the low smoking intensity and short historical trend in tobacco use in developing countries like Mongolia, the results from the indirect method may be less reliable. Hence our paper will contribute to the international literature with results based on local smoking survey data and appropriate risk estimates. Quantitative measures of the smoking-attributable lung cancer burden contribute to a better understanding of the effect of smoking on health in Mongolia, which may be used to assist in setting priorities for public health prevention policy. Therefore, we aimed to estimate the burden associated with lung cancer mortality and lung cancer incidence stratified by year and gender, and to quantify the population attributable fraction of the lung cancer burden for tobacco smoking in 2016 in Mongolia.

## Materials and methods

Population-based registry data on lung cancer incidence and mortality from the National Cancer Registry were used to estimate the lung cancer burden in disability-adjusted life years (DALYs) loss. Next, we used ever-smoking prevalence data from the population-based STEP survey performed in 2013 to estimate the population-attributable fraction (PAF) of lung cancer-related death attributable to ever-smoking in Mongolia, adding the PAFs of current smoking and former smoking[12][13][14]. The pooled estimates of relative risks of lung cancer-related death of “ever-smoking”; current smokers and former smokers compared to never smokers, were taken from region-specific cohort studies [21][22][23].

Finally, we summarized the PAF of lung cancer-related DALYs lost attributable to “ever-smoking” by gender in Mongolia.

All calculations were performed separately on single-year age groups before being added to compute the overall DALYs. Where necessary, data for five-year age groups were interpolated into single-year age groups and missing observation calendar years were imputed using spline functions [24]. To avoid working with age categories with very small numbers, for those over 65 years of age, a single aggregated category of 65+ was used.

Softwares used were MS Excel (Version 2010, Microsoft Corp, USA), STATA (Version 2014, StataCorp, USA) and the DisMod tool, developed by the Global burden of Diseases group [25].

### Data sources

#### Lung cancer incidence and mortality data

The National Cancer Registry (NCR) is a patient-based cancer registry database managed by the National Cancer Center in Mongolia. This registry collects data on newly diagnosed cancer cases and cancer deaths in Mongolia, including data from nineteen general hospitals in 21 provinces, five regional Diagnosis Centres, and all three National Clinical hospitals in the capital city. Incident cases of lung cancer and lung cancer deaths are recorded using ICD-10 codes according to (C34) cancer site [26]. Data was available in aggregated form by gender and five-year age groups for the period 2007 to 2016.

The one-year survival rate changed from 75% in 2007 to 93% in 2016. More than 90% of incident cases were diagnosed at the latest stage (T3 and T4) in 2016.

#### Smoking prevalence in Mongolia

A cross-sectional periodic STEP survey was first conducted in 2006 and later in 2009 and 2013[12][13][14]. A multistage, random cluster sampling method was adopted, targeting a set of country representative sites, totalling 42 (21 urban and 21 rural) in 2006, 60 (28 urban and 32 rural) in 2009 and 65 (32 urban and 33 rural) in 2013. Randomly selected adults aged 15–64 participated in the survey at each site. Data was available in aggregated form by gender and five-year age groups for 15–64 years old. The current smoking prevalence was stable over the past three STEP survey rounds that reported rates of 24.2%-24.8% between 2006 and 2013. Former smoking prevalence was reported separately from never smoking prevalence only for the STEP survey in 2013 which we hence used in our calculations.

#### Relative risk (RR) of tobacco smoking

Due to a lack of country specific studies on the association between tobacco smoking and lung cancer deaths, priority was given to recent large population-based prospective cohort studies from well-studied Asian countries (Japan, Korea and China). (S1 File). Data were extracted from the original studies [21][22][23], which were identified via a recent systematic reviews [8][27].

Inclusion criteria of studies were:

- Large sample size (N > 100,000) and population-based cohort study;
- RRs of lung cancer-related death presented for current smokers and for former smokers as compared to never smokers;
- Availability of RR estimates for men and women separately with 95% confidence intervals (CI);
- Length of follow-up time should be sufficient (≥5 years)
- Smoking behaviour should be comparable to Mongolia, i.e. no influence of bidi smoking; (S1 File).

We combined three cohort studies satisfying these inclusion criteria (Table 1) and pooled RRs of lung cancer death for current smokers and former smokers compared to never smokers separately for both gender using a random effects model to allow for differences among studies.

**Table 1:** Relative risk of cigarette smoking for lung cancer related deaths according to smoking category by gender in Japan, Korea and China.

References	Descriptions of the cohorts (NAME; Period; Size; Age range)	Follow-up (years)	RR of lung cancer-related death (CI 95%)			
			Current smoker versus never smoker		Former smoker versus never smoker	
			Men	Women	Men	Women
Jee et al., 2004	Korean Cancer Prevention Study (***KCPS***) cohort 1993–2001. n = 833,608 men; n = 473,667 women 30–95 years.	8	4.6 (4.0–5.3)	2.5 (2.0–3.1)	2.2 (1.9–2.6)	1.7 (1.2–2.3)
Chen et al., 2015	China Kadoorie Biobank study 2004–2008 n = 210,259 men; n = 302,632 women 30-79 years.	7	2.51 (2.37–2.66)	2.28 (1.84–2.81)	1.53 (1.28–1.81)	-
Ando et al., 2003	Japan Collaborative Cohort (JACC) 1988–2003. n = 46,654 men; n = 64,327 women 40–74 years.	15	4.46 (3.10–6.41)	3.58 (2.24–5.73)	2.38 (1.61–3.51)	2.56 (1.12–5.83)

### Estimation of population attributable fraction of DALYs lost due to lung cancer

#### Estimation of disability-adjusted life years (DALYs)

We measured disability-adjusted life years (DALYs) as the number of years lost due to disability and mortality, as proposed by Murray [28].

Years lived with disability (YLDs) and years of life lost (YLLs) were estimated separately by single-year age group, gender and year, based on lung cancer incidence and mortality between 2007 and 2016. DALYs were then estimated according to age, gender and year by adding the YLDs and YLLs.

#### Estimation of population attributable fraction (PAF)

The PAF was calculated based on the prevalence of smoking categories and the RR of lung cancer-related death for smoking exposures by Levin’s formula for multiple categories (K), as proposed by Hanley: [29][30]

$$PAF(\%)=\frac{\sum_{k=1}^{k}P_{k}({RR}_{k}-1)}{\sum_{k=1}^{k}P_{k}({RR}_{k}-1)+1}\;x\;100,\;k=1,2$$

Where P is the smoking prevalence, RR is the relative risk of lung cancer-related deaths for smoking categories and 2 is the number of categories in smoking exposure, namely current and former smokers.

We calculated the smoking attributable fractions of lung cancer DALYs using the following formula:

AttributableDALYs=ObservedDALYs*PAF

### Uncertainty analysis

We estimated probabilistic sensitivity analysis for the PAF by calculating the outcomes under the key parameters using the lower and upper limits of the 95% confidence intervals (CI) for current and former smoking prevalence and for the pooled RRs estimates for current and former smokers versus never smokers. For this purpose, we performed Monte Carlo simulations (n = 2000), varying all parameters separately for men and women and the estimates of the PAFs were repeatedly calculated from the randomly drawn set of input values [31]. Finally, extraction of the 2.5 and 97.5 percentiles from the 2000 simulation samples allowed 95% uncertainty ranges to be established, based on the interquartile range (IR).

## Results

A total of 3,670 lung cancer cases and 3,203 lung cancer fatalities were registered in Mongolia from 2007 to 2016 (S2 File). Lung cancer incidence and lung cancer mortality rates per 100,000 persons fluctuated over time for both sexes in Mongolia. In general, men suffered more from lung cancer, with rates being four to five times higher than for women.

Over a period of ten years, lung cancer accounted for the loss of more than 63,000 DALYs in Mongolia, 98% of which were due to YLLs (Table 2). Of these, more than 48,000 DALYs were lost by men (76%) and about 15,000 by women (24%). Overall, total YLDs, YLLs and DALYs fluctuated over time.

**Table 2:** Lung cancer related YLD, YLL, DALY and DALY rate in Mongolia 2007–2016.

*Year*	*Men*			*Women*			*Total*			
	*YLD*	*YLL*	*DALY*	*YLD*	*YLL*	*DALY*	*YLD*	*YLL*	*DALY*	DALY rate*
2007	56	4,047	4,103	17	1,586	1,603	73	5,633	5,706	218
2008	81	4,742	4,824	19	1,342	1,361	101	6,084	6,185	232
2009	72	3,959	4,031	21	1,152	1,173	94	5,111	5,205	192
2010	86	4,605	4,691	18	1,404	1,421	103	6,009	6,112	221
2011	87	4,912	4,999	19	1,255	1,274	106	6,167	6,273	223
2012	76	4,532	4,608	21	1,393	1,414	97	5,925	6,022	210
2013	95	5,267	5,362	24	1,433	1,457	119	6,700	6,819	233
2014	96	5,521	5,617	23	1,794	1,817	120	7,315	7,434	248
2015	91	5,188	5,279	23	1,487	1,510	114	6,675	6,789	222
2016	99	4,975	5,074	28	1,572	1,600	127	6,547	6,674	214
***Total***	***840***	***47*,*747***	***48*,*587***	***215***	***14*,*417***	***14*,*632***	***1*,*055***	***62*,*164***	***63*,*219***	***221***

YLDs, years lost due to disability; YLLs, years lost due to mortality; DALYs, disability-adjusted life years lost;

*per 100,000 population.

The estimates of the PAFs by gender and smoking categories, with 95% IR are summarized in Table 3. We found a pooled RR of 3.67 (95% CI = 2.26–5.97) for current smoker versus never smoker and 1.96 (95% CI = 1.47–2.61) for former smoker versus never smoker for men. These RRs were lower for women, especially for former smokers.

**Table 3:** Smoking prevalence, PAF and smoking-attributable DALYs loss related to lung cancer burden in Mongolia, 2016.

Smoking status	Variables	Men (95% CI/IR)	Women (95% CI/IR)	Total (95% CI/IR)
Current smokers	Smoking prevalence	45.4 (42.6–48.3)	4.5 (3.3–6.0)	24.8 (23–26.8)
	Pooled RR*	3.67 (2.26–5.97)	2.52 (2.09–3.04)	3.14 (2.38–4.13)
	PAF (%)	54.72 (39.4–70.30)	6.45 (3.46–9.65)	34.33 (25.58–43.71)
	DALY loss	2785 (2024–3520)	103 (54–155)	2322 (1718–2910)
Former smokers	Smoking prevalence	18.00 (15.4–21.0)	3.40 (2.6–4.4)	10.6 (9.2–12.2)
	Pooled RR*	1.96 (1.47–2.61)	1.80 (1.33–2.43)	1.92 (1.56–2.38)
	PAF (%)	14.79 (6.81–22.91)	2.61 (0.57–4.73)	8.76 (4.79–12.83)
	DALY loss	745 (365–1112)	42 (8–76)	591 (340–864)
Total	PAF (%)	58.1 (43.1–72.2)	8.9 (4.1–13.5)	38.4 (28.7–48.2)
	DALY loss	2946 (2158–3752)	139 (65–216)	2589 (1907–3226)

RR, relative risk, CI from meta-analysis; PAF, population attributable fraction, IR from PSA; DALY, disability-adjusted life years lost, IR from PSA;

*Age adjusted

Overall, the PAF of lung cancer-related deaths attributable to ever-smoking was estimated at 58.1% (95% IR = 43.1%-72.2%) for men and 8.9% (95% IR = 4.1% -13.5%) for women. Tobacco smoking was responsible for about 2946 DALY lost (95% IR = 2158–3752) for men and 139 DALY lost (95% IR = 65–216) for women in 2016 in Mongolia.

## Discussion

Our study provides a new estimate of the effect of ever-smoking prevalence on the lung cancer related disease burden in Mongolia. Lung cancer accounted for the loss of more than 63,000 DALYs in Mongolia between 2007 and 2016. We found the PAF for lung cancer deaths attributable to ever-smoking in 2016 was 38.4% (95% IR = 28.7%-48.2%). This implied that nearly 2600 DALYs (95% IR = 1907–3226) lost from lung cancer were attributable to active tobacco smoking in 2016 in Mongolia.

The differences between men and women were striking. While nine times more men smoke than women, the estimated PAF to ever-smoking was more than six times higher for men than for women. Our sensitivity analysis revealed that the PAF for lung cancer deaths attributable to ever-smoking was substantially higher for men than women when combined confidence intervals of the pooled relative risks and smoking prevalence.

Despite using different methods our results are comparable to those of similar studies conducted in other Asian countries. The attributable fraction of smoking to lung cancer death was 50% for men and 16% for women in China, 53.3% for men and 5.2% for women in Korea, 52.2% for men and 11.8% for women in Japan[32][33]. This reveals that the differences between men and women are more pronounced in Mongolia than in some other Asian countries. In contrast, according to international studies the estimated proportion of tracheal, bronchus and lung cancer death rate attributable to tobacco was higher than our results, for Mongolia, it was 91% for men and 74% for women over age 30 [3]. Moreover, in the GBD study the PAF of tracheal, bronchus and lung cancer DALYs lost to smoking was 82.2% in 2015[2]. These findings were much higher than our results, especially for women. The striking difference is explained by the high number of accumulated hazards of smoking as used in the GBD and taken from the reanalysis of American Cancer Society CPS-II data and by the use of the Peto indirect method to estimate smoking prevalence from lung cancer mortality applied in the GBD study [34]. This indirect method uses the lung cancer mortality for a known reference group of smokers as a proxy to estimate smoking prevalence in the study population [18]. In contrast, in our analysis, we had access to direct estimates of smoking prevalence from a representative surveys.

Current smokers in Mongolia have a different smoking pattern than Western populations, which explains the differences between these populations [35]. (S3 File) According to the STEP survey from 2009 to 2013, in Mongolia the mean number of cigarettes smoked per day has increased from 8.9 to 10.3 and the mean duration of smoking has increased from 16.1 to 21.2 years for men [12][13][14]. Consequently, the average pack-year has increased from 7 to 11 during this period. Likewise, for women, the mean number of cigarettes smoked per day has fallen from 7 to 6.4 while the mean duration has increased from 13 to 14.4. Therefore, for women in Mongolia the average pack-year has remained at around only 4.6. In contrast, in the US most current smokers were life-long cigarette smokers with a mean consumption of twenty cigarettes per day [19]. When smoking prevalence and intensity of smoking habits continue to increase in Mongolia, the burden of tobacco smoking will increase considerably, especially in men.

Various risk factors in addition to tobacco smoking have been identified as determinants of the lung cancer burden [1]. Outdoor air pollution is a serious public health problem in Mongolia, especially in its capital city, where the annual average concentration of particular matter (PM) was substantially higher than National and International air quality standards [36]. A conservative estimate found that about 40% of (95% CI, 17–56%) of lung cancer deaths were attributable to outdoor air pollution in Ulaanbaatar city. [37] Moreover, approximately half of Mongolian households reside in a ger (a traditional dwelling). People living in gers primarily burn biomass fuel and coal for cooking, heating and other domestic needs, and are exposed to indoor smoke although a ger will usually have some kind of chimney installed. Studies indicate that evaluated PM exposures and indoor air pollution were associated with significant increases in lung cancer mortality in China [38][39][40]. The interactions of multiple risk factors and how they affect the lung cancer risk are important to quantify the lung cancer burden in Mongolia. This study is one of a number of studies needed, given the current lack of local studies on risk factor epidemiology and specific exposure related risks.

We did not explicitly calculate the burden from second hand smoke exposure in our study because insufficient data was available during our study year. According to the last STEP survey results presented in 2013, half of all women and a third of all men in Mongolia were exposed to second hand smoke at home [14]. So for women, other causes of lung cancer are more prominent than active smoking. Passive smoking maybe one of these causes.

A strength of our study was its use of detailed epidemiological data: country-specific patient-level registry data with ten-year period trends and country representative STEP survey reports provided access to precise numbers for smoking prevalence by gender, year and smoking categories. We believe this study will provide a foundation for illustrating the impact of smoking exposure on population health separately for men and women in Mongolia.

Our study has some limitations. The smoking exposures estimate focused only on active tobacco smoking and did not include passive tobacco smoking exposure, which might underestimate the smoking attributable proportion of the lung cancer burden. Secondly, in the absence of country-specific smoking RR for diseases, we used the RR from neighbouring or nearby countries such as Japan, Korea and China. Smoking habits may be different between Mongolia and these countries, thus the effect of tobacco smoking may have been somewhat overestimated in our study, when smoking in Mongolia is less than in the countries used. While the current study presents DALYs lost through lung cancer, smoking also increases the risk of COPD, ischemic heart disease and stroke, adding to the burden of disease. Because RRs of cause-specific mortality associated with tobacco smoking were not available in Mongolia, we were unable to estimate smoking-related diseases DALY loss attributable to tobacco smoking. Using the set of RRs, the magnitude of tobacco smoking risk on population health in Mongolia is likely to be even larger than that estimated in this study. Despite some limitations mentioned above, a country-specific estimate of the PAF of lung cancer stratified by gender and smoking categories is an important starting point for future epidemiological and (health) economic studies, i.e. in line with global initiatives such as WHO-FTCT, Universal Health Coverage and UN Sustainable Development goals.

Number of policy documents has been published in Mongolia with the aim to reduce exposure to major risk factors including tobacco smoking. Yet, most programmes are largely focused on population-level policies [41]. The Tobacco Control Law was revised in 2012, with major changes including rules for the sale of tobacco to and by people under the age 21, restrictions on smoking in public areas, and the prohibition of cigarette product advertising [42]. In contrast, according to the WHO-MPOWER report, community and individual- level prevention policies are lacking such as cessation treatment, nicotine replacement treatment and quit lines [43].

In the meantime in Mongolia almost half of men are currently smokers and half of women are exposed to second hand smoke at home [44]. Focusing on male populations through community and individual-level interventions may also help protect non-smokers, children, pregnant women and others from active and passive smoke exposure in Mongolia.

## Conclusions

Lung cancer accounted for the loss of over 63,000 DALYs between 2007 and 2016 in Mongolia. A considerable health loss may be prevented with an effective anti-smoking policy. In Mongolia, more than one third of lung cancer-related DALY loss is attributable to active smoking, and thus is potentially preventable. Furthermore, due to the high gender difference found in the effects of smoking, a gender-specific tobacco control policy seems needed to curb the increasing trend in smoking-related diseases burden in Mongolia.

## Supporting information

S1 File Relative risks of lung cancer death associated with tobacco smoking. (DOCX)

S2 File Lung cancer incidence and lung cancer mortality by gender in Mongolia. (DOCX)

S3 File Adult population smoking daily by gender, 2015 (or nearest year). (DOCX)
