# Supplementary material for: Smoking-attributable burden of lung cancer in Mongolia a data synthesis study on differences between men and women
Source: PLoS One. 2020 Feb 14;15(2):e0229090. doi: 10.1371/journal.pone.0229090 (PMC7021290; doi:10.1371/journal.pone.0229090)
Supplement: S1 File — (DOCX) [file pone.0229090.s001.docx]

# **APPENDIX**

S1 Table 1: Relative risks of lung cancer death associated with tobacco smoking

| Country | Sex | RR for  Current  smoker | RR for  Fomer  smoker | | Comments |
| --- | --- | --- | --- | --- | --- |
| Mongolia | Male | 3.67 (2.26-5.97) | 1.96 (1.47-2.61) | | Our estimated RR. |
|  | Female | 2.52 (2.09-3.04) | 1.80 (1.33-2.43) | |  |
| China/Korea | Male | 3.56(2.45-5.16) | | | [1] |
|  | Female | 3.34(2.29-4.86) | | |  |
| Japan | Male | 4.12(3.49-4.87) | | |  |
|  | Female | 3.15(2.70-3.68) | | |  |
| Taiwan | Male | 3.7(2.1-6.6) | | Not available | [2] |
|  | Female | 3.6(1.0-12.2) | | Not available |  |
| Asian pooled analysis | Male  Birth cohort < 1920  Birth cohort 1920-1929  Birth cohort ≥ 1930 | 3.38(2.25-5.07)  4.74(3.56-6.32)  4.80(3.71-6.19) | 3.00(2.24-4.02)  3.77(2.94-4.84)  4.09(3.26-5.15) | | [3] |
|  | Female  Birth cohort < 1920  Birth cohort 1920-1929  Birth cohort ≥ 1930 | 2.94(2.33-3.71)  4.17(3.25-5.35)  3.53(2.99-4.16) | 2.87(2.22-3.71)  3.85(3.04-4.87)  3.21(2.77-3.72) | |  |
| USA | Male | 25.3(21.10-30.34) | 7.60(6.32-9.15) | | [4] |
|  | Female | 12.62(11.13-14.31) | 3.77(3.25-4.38) | |  |

References:

[1] W. Zheng, D. F. McLerran, and B. A. Rolland, “Burden of Total and Cause-Specific Mortality Related to Tobacco Smoking among Adults Aged ≥45 Years in Asia: A Pooled Analysis of 21 Cohorts,” *PLoS Med.*, vol. 11, no. 4, p. e1001631, Apr. 2014.

[2] K.-M. Liaw and C.-J. Chen, “Mortality attributable to cigarette smoking in Taiwan: a 12-year follow-up study,” *Tob. Control*, vol. 7, pp. 141–148, 1998.

[3] J. J. Yang *et al.*, “Tobacco Smoking and Mortality in Asia: A Pooled Meta-analysis,” *JAMA Netw. open*, vol. 2, no. 3, p. e191474, Mar. 2019.

[4] M. J. Thun *et al.*, “50-Year Trends in Smoking-Related Mortality in the United States,” *N. Engl. J. Med.*, vol. 368, no. 4, pp. 351–364, Jan. 2013.
