# Supplementary material for: Smoking-attributable burden of lung cancer in Mongolia a data synthesis study on differences between men and women
Source: PLoS One. 2020 Feb 14;15(2):e0229090. doi: 10.1371/journal.pone.0229090 (PMC7021290; doi:10.1371/journal.pone.0229090)
Supplement: S2 File — (DOCX) [file pone.0229090.s002.docx]

# **APPENDIX**

| S2 Table 2: Lung cancer incidence and lung cancer mortality by gender in Mongolia | | | | | | | | | | | |
| --- | --- | --- | --- | --- | --- | --- | --- | --- | --- | --- | --- |
| Year | Incidence | | | | |  | Mortality | | | | |
|  | Men | |  | Women | |  | Men | |  | Women | |
|  | Cases | Rates* |  | Cases | Rates* |  | Cases | Rates* |  | Cases | Rates* |
| 2007 | 196 | 15 |  | 60 | 4 |  | 217 | 17 |  | 68 | 5 |
| 2008 | 282 | 22 |  | 68 | 5 |  | 255 | 20 |  | 65 | 5 |
| 2009 | 251 | 19 |  | 75 | 5 |  | 220 | 17 |  | 52 | 4 |
| 2010 | 297 | 22 |  | 62 | 4 |  | 248 | 18 |  | 67 | 5 |
| 2011 | 302 | 22 |  | 68 | 5 |  | 267 | 20 |  | 56 | 4 |
| 2012 | 264 | 19 |  | 74 | 5 |  | 226 | 16 |  | 66 | 4 |
| 2013 | 331 | 23 |  | 85 | 6 |  | 275 | 19 |  | 64 | 4 |
| 2014 | 334 | 23 |  | 81 | 5 |  | 285 | 19 |  | 72 | 5 |
| 2015 | 319 | 21 |  | 80 | 5 |  | 283 | 19 |  | 71 | 5 |
| 2016 | 344 | 22 |  | 97 | 6 |  | 279 | 18 |  | 67 | 4 |
| Total | 2920 | 21 |  | 750 | 5 |  | 2555 | 18 |  | 648 | 4 |
| *Per 100,000 population | | | | | | | | | | | |
