# Supplementary material for: Smoking-attributable burden of lung cancer in Mongolia a data synthesis study on differences between men and women
Source: PLoS One. 2020 Feb 14;15(2):e0229090. doi: 10.1371/journal.pone.0229090 (PMC7021290; doi:10.1371/journal.pone.0229090)
Supplement: S3 File — (DOCX) [file pone.0229090.s003.docx]

# **APPENDIX**

S3 Figure 1: Adult population smoking daily by gender, 2015 (or nearest year)

*Source: OECD Health Statistics 2017.*
